# Mapping the energy landscape of protein–ligand binding *via* linear free energy relationships determined by protein NMR relaxation dispersion†

**DOI:** 10.1039/d0cb00229a

**Published:** 2020-12-23

**Authors:** Olof Stenström, Carl Diehl, Kristofer Modig, Ulf J. Nilsson, Mikael Akke

**Affiliations:** Division of Biophysical Chemistry, Center for Molecular Protein Science, Department of Chemistry, Lund University Box 124 SE-22100 Lund Sweden mikael.akke@bpc.lu.se; Centre for Analysis and Synthesis, Department of Chemistry, Lund University Box 124 SE-22100 Lund Sweden

## Abstract

Biochemical signaling is mediated by complexes between macromolecular receptors and their ligands, with the duration of the signal being directly related to the lifetime of the ligand–receptor complex. In the field of drug design, the recognition that drug efficacy *in vivo* depends on the lifetime of the drug–protein complex has spawned the concept of designing drugs with particular binding kinetics. To advance this field it is critical to investigate how the molecular details of designed ligands might affect the binding kinetics, as well as the equilibrium binding constant. Here we use protein NMR relaxation dispersion to determine linear free energy relationships involving the on- and off-rates and the affinity for a series of congeneric ligands targeting the carbohydrate recognition domain of galectin-3. Using this approach we determine the energy landscape and the position of the transition state along the reaction coordinate of protein–ligand binding. The results show that ligands exhibiting reduced off-rates achieve this by primarily stabilizing the bound state, but do not affect the transition state to any greater extent. The transition state forms early, that is, it is located significantly closer to the free state than to the bound state, suggesting a critical role of desolvation. Furthermore, the data suggest that different subclasses of ligands show different behavior with respect to these characteristics.

## Abstract 2

NMR relaxation dispersion experiments reveal linear free energy relationships relating the binding constants to the lifetimes of protein–ligand complexes, showing that the transition state is located close to the free state.

## Graphical abstract

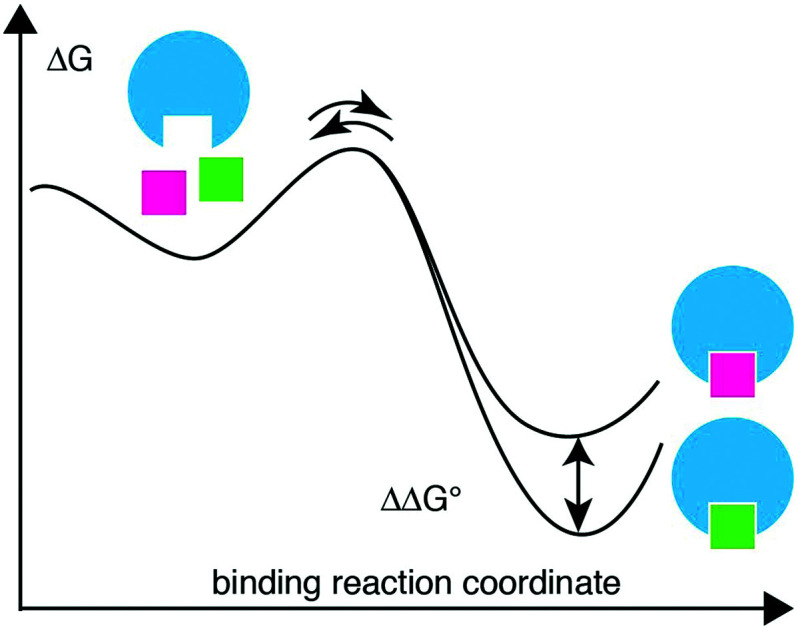

## Introduction

Transient formation of complexes between macromolecular receptors and their ligands is central to biochemical signaling. Thus, the time scale of target protein activation or inhibition is of central interest both from a fundamental physiological perspective and in the field of drug design. The important role of binding kinetics, *i.e.*, the rates of ligand association with and dissociation from target proteins, is increasingly being recognized in the field of drug design.^1–5^ In practical terms, the life time of a ligand–receptor complex is most often a better predictor of *in vivo* efficacy than is binding affinity. Despite the importance of these effects, current understanding of the molecular determinants controlling binding rates remains incomplete. A central question is how differences among ligands in their binding rates are explained by the relative stabilization or destabilization of the bound state and transition state.^2^ Here we use protein NMR relaxation dispersion experiments to address this question by mapping the energy landscape of ligand binding and study how it varies among a set of related ligands with variable affinity for the pharmaceutical target protein, galectin-3.

**Fig. 1:**
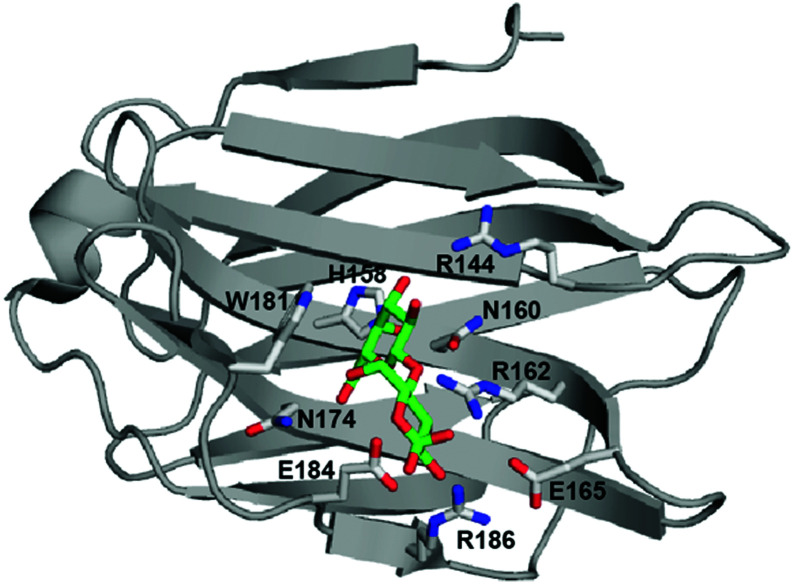
Crystal structure of lactose-bound galectin-3C (PDB 3ZSJ). Side chains coordinating the ligand are shown in stick representation. Lactose is colored green (carbon atoms) and red (oxygen atoms). The figure was prepared using PyMOL (Schrödinger, LLC).

Galectins define a family of carbohydrate binding proteins with a highly conserved carbohydrate recognition domain, which preferentially binds β-galactoside-containing glycans composed of *N*-acetyllactosamine (Galβ1–4GlcNAc; LacNAc).^6,7^ Galectin-3 has been implicated in cell growth, cell differentiation, cell cycle regulation, and apoptosis, and therefore constitutes a target for the treatment of inflammation and cancer.^8,9^ Monovalent carbohydrate–galectin interactions of mono- or disaccharides are relatively weak, with dissociation constants on the order of 0.1–10 mM and short lifetimes. The weak binding of saccharides is partly due to their polarity, frequent lack of charges, and limited hydrophobicity, which reduces their potential for forming strong protein–ligand interactions and makes the design of high-affinity carbohydrate mimicking inhibitors quite challenging. These ligand properties are reflected by the carbohydrate binding site of galectins, as exemplified by galectin-3, which is located in a shallow and water-exposed groove of a six-stranded β-sheet (Fig. 1).

Recent studies of galectin-3 have shown that the carbohydrate binding site is preorganized to recognize a sugarlike framework of oxygens,^10^ which has served as the starting point for successful design initiatives leading to inhibitors with dissociation constants in the nanomolar range.^11,12^ Residues defining the carbohydrate binding site are highly conserved among members of the galectin family.^6^ β-Galactoside ligands interact through hydrogen bonds with residues H158, N160, R162, N174, E184 and R186, while W181 stacks against the galactose moiety (Fig. 1; residue numbering according to the galectin-3 sequence).

Recent advances in the design and application of Carr–Purcell–Meiboom–Gill (CPMG) relaxation dispersion experiments^13–15^ have demonstrated their great utility for studying conformational or chemical exchange, including the specific case of ligand binding.^16–20^ This powerful method can provide information on the rate of conformational exchange (*k*_ex_), the populations of the exchanging states (*p*), as well as the differences between these in chemical shifts (Δ*δ*_CPMG_), which carry structural information. Here we apply this approach in a comparative study of a series of congeneric ligands in order to investigate the energy landscape of the binding reaction using the concept of linear free energy relationships.^21–23^

## Results and discussion

**Fig. 2:**
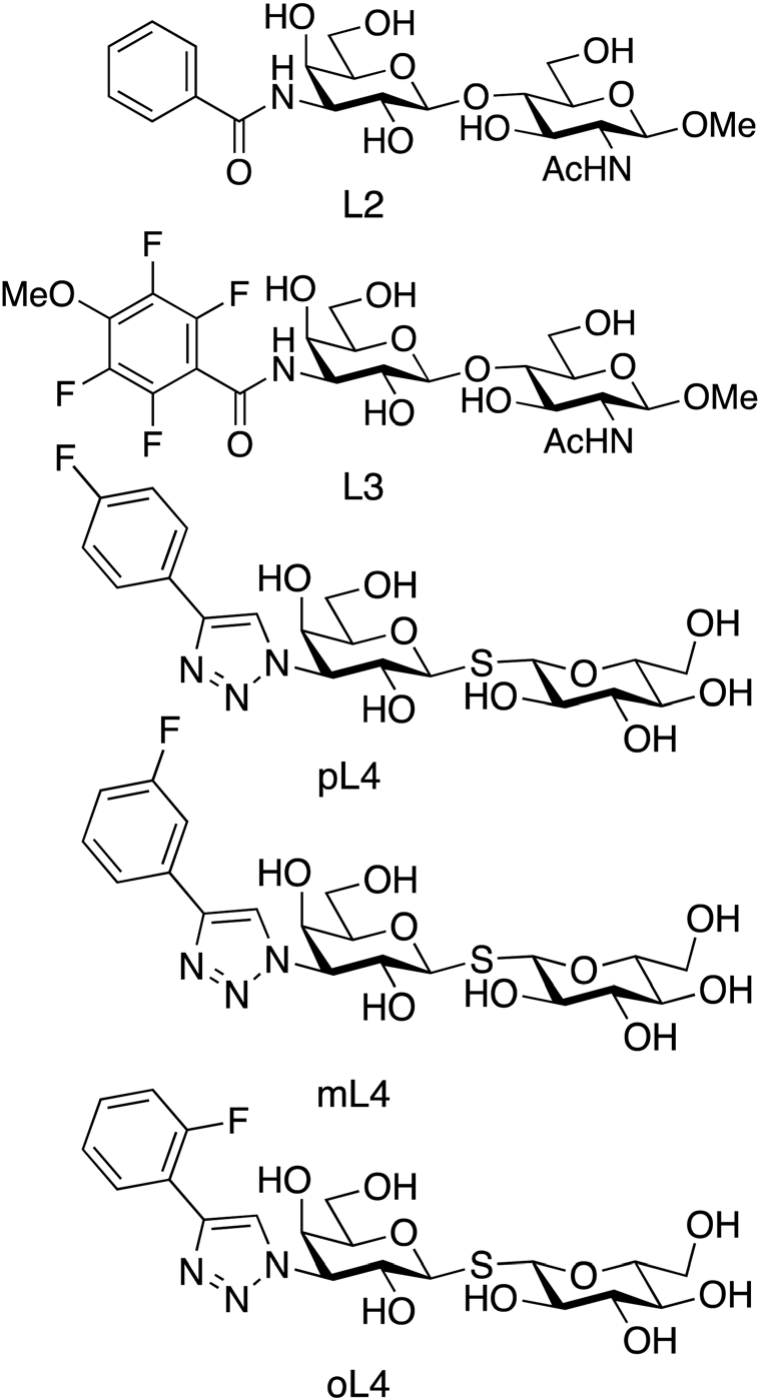
Chemical structures of the 5 ligands studied here: (L2) 3′-benzamido-*N*-acetyllactoseamine; (L3) 3′-(4-metoxy-2,3,5,6-tetrafluorobenzamido)-*N*-acetyllactoseamine; (pL4, mL4, oL4) *para*-, *meta*-, and *ortho*-fluoro-phenyltriazolyl-galactosylthioglucoside, respectively.

We investigated the relationship between ligand residence time and affinity for the carbohydrate-recognition domain of galectin-3 (galectin-3C) using ^15^N CPMG relaxation dispersion. We used a set of congeneric inhibitors that span one order of magnitude in affinity: 3′-benzamido-*N*-acetyllactosamine (denoted L2), 3′-(4-methoxy-2,3,5,6-tetrafluorobenzamido)-*N*-acetyllactosamine (denoted L3) and *para*-, *meta*-, and *ortho*-fluoro-phenyltriazolyl-galactosylthioglucoside (denoted pL4, mL4 and oL4, respectively),^24–26^ see Fig. 2.

These ligands all bind *via* the canonical interactions mentioned above, together with additional interactions between the phenyl moiety of the ligand and arginine side chains, backbone amide groups, and hydrophobic groups in the protein. The ligands can be divided into two subclasses related to the carbohydrate scaffold and phenyl substitutions: the L4 variants have a non-substituted glucose ring, whereas L2 and L3 contain methoxy and acetamido moieties; the L4 variants contain a fluoro-phenyltriazole group, whereas L2 and L3 both contain a benzamido group (Fig. 2).

### Ligand binding kinetics measured by ^15^N NMR relaxation dispersion experiments reveal variable off-rate constants

**Fig. 3:**
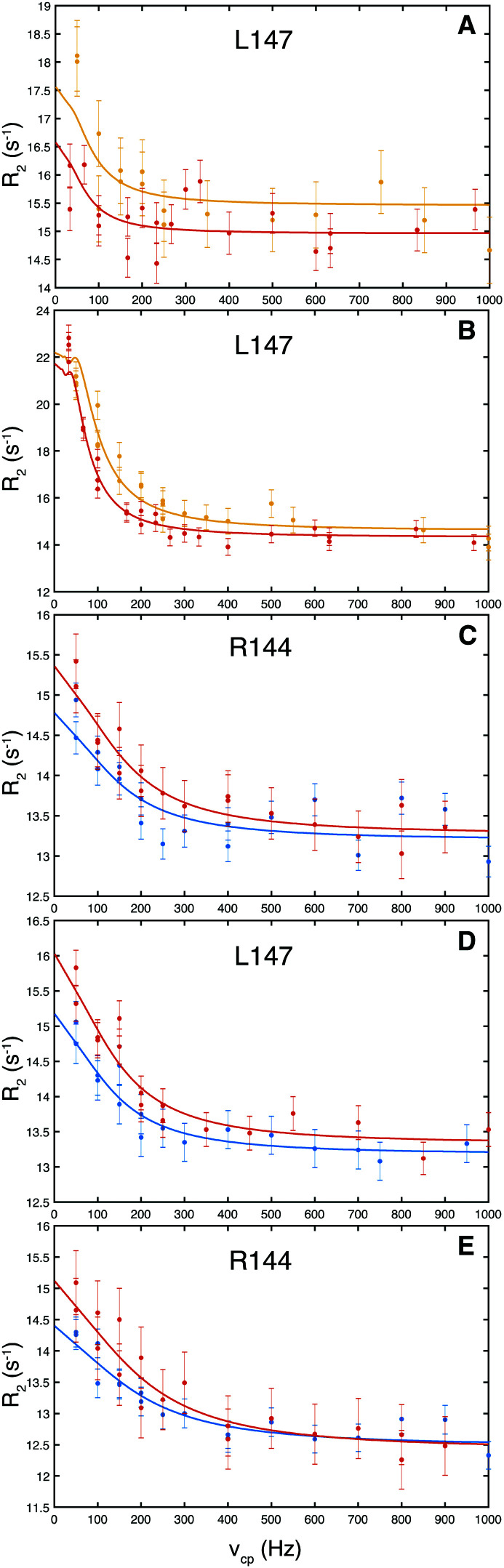
Representative ^15^N CPMG relaxation dispersion profiles for residues in galectin-3C bound to: (A) L2, (B) L3, (C) pL4, (D) mL4, (E) oL4. Data for L2- and L3-bound galectin-3C were obtained at static magnetic field strengths of 14.1 T and 18.8 T, represented by red and orange symbols, respectively, while data for L4-bound galectin-3C were obtained at 11.7 T (blue) and 14.1 T (red).

We measured equilibrium exchange between free and ligand-bound states of galectin-3C using ^15^N CPMG relaxation dispersion experiments^15^ acquired on near-saturated samples at two static magnetic field strengths. Representative relaxation dispersion profiles for the 5 complexes are shown in Fig. 3 (the ESI,† includes ^1^H–^15^N HSQC spectra of all complexes and all relaxation dispersion data; see Fig. S1–S6). We analyzed the relaxation dispersion profiles using a two-state exchange model (eqn (1)–(4)).^18,27,28^ Based on initial residue-specific fits we identified two groups of residues in each complex that showed either slow to intermediate exchange (*k*_ex_ ≈ 120–1200 s^−1^) or faster exchange (*k*_ex_ > 4000 s^−1^). Notably, the more slowly exchanging residues are located in the ligand binding site, whereas the faster exchanging ones are located on the opposite side of the β-sandwich (Fig. S7, ESI†), leading to the tentative interpretation that the slower exchange is due to ligand binding and release, whereas the faster group undergoes conformational exchange unrelated to binding. All residues within a given group were then fitted jointly to yield global exchange parameters, *k*_ex_ and *p*, and residue-specific Δ*δ*_CPMG_ (ESI,† Table S1 lists all residues included in the fit for the two groups of each complex together with the fitted parameters). In the following, we focus on the exchange related to ligand binding and release.

**Fig. 4:**
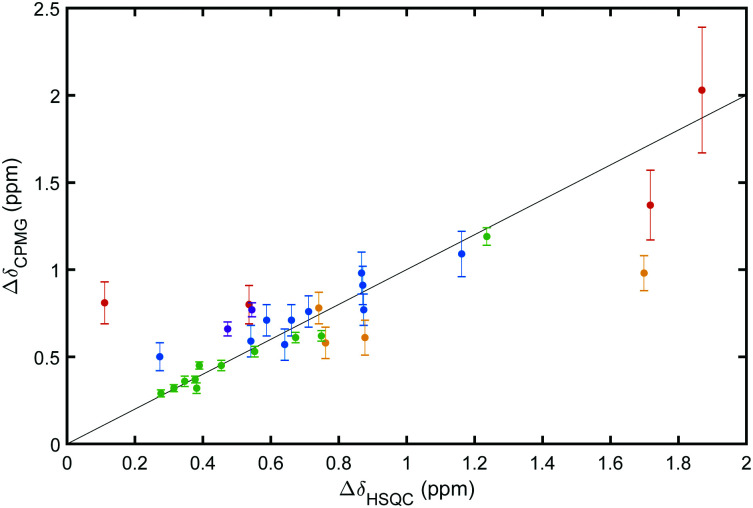
Comparison of chemical shift differences determined from CPMG dispersion experiments (Δ*δ*_CPMG_) and HSQC spectra (Δ*δ*_HSQC_) for galectin-3C bound to: purple, L2; green, L3; red, pL4; blue, mL4; yellow, oL4. The straight line with a slope of 1 is drawn to guide the eye.

The resulting values of Δ*δ*_CPMG_ and *k*_ex_ indicate that all residues reporting on ligand binding exhibit intermediate to slow exchange, which vouches for high accuracy of Δ*δ*_CPMG_ when determined from data obtained at two static magnetic field strengths,^29^ as is the case here. The resulting chemical shift differences, Δ*δ*_CPMG_, show a high level of correlation with the those measured between the ^1^H–^15^N HSQC spectra of apo (ligand-free) and ligand-bound galectin-3C, Δ*δ*_HSQC_ (Fig. 4), demonstrating that the observed exchange indeed reflects ligand-binding kinetics. Apparent outliers in Fig. 4 are observed for complexes with few data points, which might be expected, because Δ*δ*_CPMG_ is generally better determined for those complexes that include a greater number of degrees of freedom of the fit, *i.e.*, a larger number of fitted residues.

The relative population of the bound state, *p*_B_, varies between 0.93–0.94 for L2 and L3, and 0.97–0.99 for the L4 complexes, in excellent agreement with values calculated from the reactant concentrations in the NMR sample and *K*_d_, determined previously by isothermal titration calorimetry (ITC) under nearly identical sample conditions.^25^Table 1 lists the determined values of *k*_off_ = *k*_ex_(1 − *p*_B_) and *k*_on_ = *k*_off_/*K*_d_ for the 5 different ligand-galectin-3C complexes. In the present case, we determined all exchange parameters directly from the relaxation dispersion data. This approach is applicable to systems exhibiting slow to intermediate exchange rates, which correspond to off-rate constants broadly in the range of 1–100 s^−1^. Significantly faster off-rate constants can likely be determined if the populations of the free and bound states, as well as the chemical shift difference between states, can be determined separately and taken as fixed values in the fitting procedure. This requires that the ligand and protein concentrations are measured accurately, *K*_d_ is known from previous experiments, and the NMR spectra of the free and bound states are available; these requirements are easily met in most cases. While the amplitude and shape of the relaxation dispersion profile depends intricately on the populations, rates, and chemical shift difference, it may be expected that off-rates can be determined for systems with dissociation constants in the range of 0.1 μM to 1 mM.

**Table 1:** Rate constants and affinities for ligand binding to galectin-3C

Ligand	*k*_off_ (s^−1^)	*k*_on_ a (10^6^ M^−1^ s^−1^)	*K*_d_ b (10^−6^ M)
L2	28 ± 9	1.6 ± 0.5	18.2 ± 0.2
L3	8 ± 1	2.4 ± 0.4	3.3 ± 0.1
pL4	13 ± 1	5.2 ± 0.8	2.5 ± 0.3
mL4	8 ± 1	3.9 ± 0.7	2.0 ± 0.3
oL4	32 ± 5	4.4 ± 1.1	7.2 ± 1.3

a *k*_on_ is determined from *K*_d_ and *k*_off_.

b Dissociation constants determined by ITC under sample conditions near-identical to those used for CPMG dispersion experiments.

We investigated the relationship between the reaction rate constants and the binding affinity. Notably, within each of the two ligand subclasses, *k*_off_ shows a trend of progressively slower rates with increasing affinity. Thus, the lifetimes of the complexes for the present series of ligands correlate with the binding affinity. However, we note that the rank order does not hold across both subclasses of ligands, *viz.* L3 and mL4, as well as L2 and oL4, have identical *k*_off_ but different *K*_d_. This deviation can conceivably be explained by the fact that the two ligand subclasses differ in key structural aspects, as explained above (Fig. 2).

### Linear free energy relationships define the energy landscape of ligand binding

**Fig. 5:**
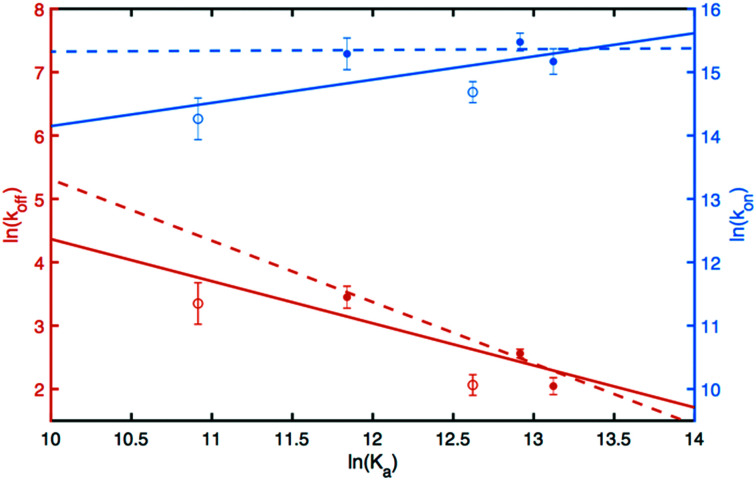
Linear free energy relationships for ligand binding to galectin-3C. Activation barriers associated with the on- and off-rates plotted as a function of the standard free energy of binding. Red, ln(*k*_off_), blue, ln(*k*_on_). Filled circles represent the L4 ligands, while open circles represent L2 and L3. The solid lines show the linear fits to the data. Note that the scales are different on the left-hand-side (ln(*k*_off_), red) and right-hand-side (ln(*k*_on_), blue) axes. The full lines represent linear fits to the full data set comprising all 5 complexes, while the dashed lines represent fits to the L4 subset of complexes.

The results can be analyzed in more detail by means of linear free energy relationships (LFER) involving the standard free energy of binding and the activation free energies for the on- and off-rate constants across the series of different ligand-protein complexes; see Experimental section for a brief description of the underlying theory (eqn (5)–(9)). The concept of LFER has a long history in physical organic chemistry^21^ and has been applied successfully to proteins.^22,30,31^ LFER can be determined by correlating the logarithms of *k*_off_, *k*_on_, and *K*_a_ = 1/*K*_d_, as shown in Fig. 5. Notably, we find that ln(*k*_off_) shows a stronger dependence on ln(*K*_a_) than does ln(*k*_on_), indicating that the different ligands stabilize the bound state to variable extents, but appear to have less effect on the transition state between the free and bound states.

### The transition state is formed early on the ligand-binding reaction coordinate

Invoking a Brønsted or Leffler type analysis,^21,22^ the slope of the LFER provides additional information on the energy landscape (eqn (8) and (9)). The slope, 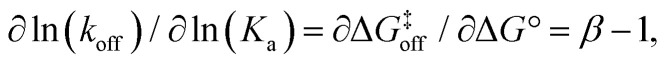 is a measure of the position of the transition state on the reaction coordinate from the free state (*β* = 0) to the bound state (*β* = 1). It should be noted that this measure rests on the assumption that the free energies of the free and bound states can be described by harmonic mean-field potentials with equal curvature. It might be expected that the bound state has a steeper free energy basin than does the free state, in which case *β* yields a lower value than the true position of the transition state. The approach is similar to *ϕ*-value analysis of protein folding kinetics.^22^ Despite the obvious limitations of this type of analysis, which projects a multi-dimensional coordinate space onto a one-dimensional reaction coordinate, it is arguably well suited to treating ligand release from a relatively exposed binding site, which is a considerably less complex process than protein folding.

The linear fit of ln(*k*_off_) *versus* ln(*K*_a_) yields a correlation coefficient of *r* = −0.84 and a slope of 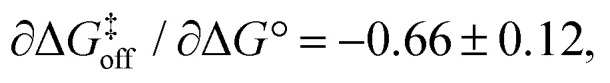 when using data for all five complexes (Fig. 5, full line). Thus, the result *β* = 0.34 indicates that the transition barrier is positioned considerably closer to the free state than to the bound state. Considering the structural differences between the two ligand subclasses, we also performed the analysis separately for the L4 subclass, which yielded *r* = −0.98, 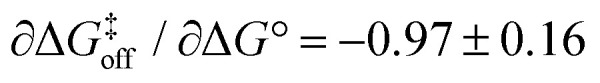 and *β* = 0.03 (Fig. 5, dashed line). In this case, it appears that the transition state forms very early along the binding reaction coordinate, suggesting that it might involve desolvation of the ligand and protein prior to the formation of coordinating interactions.

Taken together, these results hint at an underlying difference in the position of the transition state between the two subgroups that might relate to their structural differences; for example, one can speculate that the difference in flexibility of the phenyltriazole (in L4) and benzamido (in L2 and L3) linkages might play a role. In this context, we also note that the majority of residues exhibiting exchange are located in proximity of the very part of the ligand that shows the largest structural variation, *i.e.*, the non-saccharide moiety. Following the Hammond postulate,^22,32^ the early transition state, observed for both the full set of 5 ligands and the L4 subset, indicates that complex formation involves structural reorganization after the binding reaction has passed the transition barrier.

## Conclusions and outlook

In conclusion, we have demonstrated the use of protein NMR relaxation dispersion to characterize the relationship between the lifetimes and binding affinities of protein–ligand complexes under equilibrium conditions and without any perturbation of the system in terms of molecular tagging or immobilization of the protein or ligand. Using a designed series of ligands with minor structural differences, this approach makes it possible to outline linear free energy relationships that provide previously elusive information on the nature of the transition state and on the relative stabilization of the bound state over the transition state. Thus, the experimental data provide important information that should be useful for guiding and benchmarking computational approaches to design drugs with desired kinetic profiles. The present results suggest that the two classes of ligands might exhibit different linear-free energy relationships. To this extent, it appears that the detailed ligand structure can have an effect on the energy landscape of ligand binding and on the position of the transition state along the reaction coordinate, with exciting prospects for ‘kinetic’ drug design. This interesting observation merits future investigations using expanded ligand series to pinpoint molecular features that might affect the transition state.

## Experimental

### Ligand synthesis

The synthesis protocols of ligands L2, L3, oL4, mL4, and pL4 have been described.^24–26^

### Protein expression and purification

Galectin-3C was expressed and purified following published protocols,^25,33^ yielding a protein stock solution of 16 mg ml^−1^ in ME-PBS buffer, consisting of 10 mM Na_2_HPO_4_, 1.8 mM KH_2_PO_4_, 140 mM NaCl, 2.7 mM KCl, pH 7.3, 2 mM ethylenediaminetetraacetic acid (EDTA), 4 mM tris(2-carboxyethyl)phosphine hydrochloride (TCEP), and 150 mM lactose. The protein stock solution was stored at 278 K.

### NMR sample preparation

^15^N-Labeled galectin-3C samples were prepared by extensive dialysis (Slide-A-Lyzer MINI Dialysis, ThermoScientific) against 5 mM 4-(2-hydroxyethyl)-1-piperazinethanesulfonic acid (HEPES) buffer to remove all lactose, followed by centrifugation at 14 000 rpm for 5 minutes to remove any aggregates. The protein concentration was determined by UV absorption at 280 nm, as described before.^33^ Ligands L2 and L3 were dissolved in dimethyl sulfoxide (DMSO) stock solutions to concentrations of 120 and 60 mM, respectively, while L4 ligands were dissolved into 7–10 mM stock solutions using the same HEPES buffer as that used in the protein samples. Ligand–galectin-3C complexes were prepared by titrating freshly sonicated stock solutions of the ligands into apo galectin-3C samples while monitoring the galectin-3C chemical shifts in the ^15^N heteronuclear single-quantum correlation (HSQC) spectrum. At the end of each titration, the total amount of added DMSO in the L2 and L3 samples was 2% (v/v). The resulting ligand–galectin-3C samples had protein concentrations of 0.4 mM (L2 and L3 complexes) or 0.8 mM (L4 complexes) and ligand concentrations of 0.61 mM (L2), 0.43 mM (L3), 0.99 mM (mL4), 1.04 mM (pL4), and 1.50 mM (oL4). Galectin-3C was 93–99% saturated with ligand in each resulting sample, as calculated from the concentrations of ligand and protein together with the value of *K*_d_ determined by ITC measurements conducted under near-identical conditions.

### NMR relaxation dispersion experiments

Backbone amide ^15^N CPMG relaxation dispersion experiments were performed at static magnetic field strengths of 14.1 T and 18.8 T for the L2 and L3 complexes, and 11.7 T and 14.1 T for the mL4, pL4 and oL4 complexes, using Varian/Agilent VNMRS DirectDrive spectrometers. The temperature was 301 ± 0.1 K. Temperature calibration was performed prior to each relaxation series using either a methanol reference sample (L2 and L3 samples) or a type-T copper-constantan thermocouple element with one electrode in an ice-water bath and the other in an NMR tube in water, positioned at the sample location inside the magnet (L4 samples). Relaxation-compensated CPMG relaxation dispersion experiments^15^ were performed using the constant-time approach^34^ covering 18–24 different refocusing frequencies, *ν*_cp_. An equilibration period of 5 ms was added prior to the CPMG refocusing pulse train to ensure that the initial magnetizations reflect the relative populations of the exchanging states.^35^ The experiments performed at 18.8 T utilized the phase cycle proposed by Yip & Zuiderweg to suppress artefacts due to off-resonance effects.^36^ Spectra were processed using NMRPipe.^37^ The processing protocol involved a solvent filter, cosine-squared apodization functions, zero filling to twice the number of increments in all dimensions, and a polynomial baseline correction in the ^1^H dimension. Relaxation rates were extracted using PINT, which employs line-shape fitting to resolve overlapped peaks.^38^ Peak intensities were evaluated using a weighted sum of Lorentzian and Gaussian line shapes. Standard deviations were determined by propagating the errors of intensities estimated from the baseplane noise. Error estimates are reported as one standard deviation.

### NMR data analysis

The relaxation dispersion data were analyzed using CPMGfit v2.22, an in-house Matlab program. Relaxation dispersion curves were fitted to the Carver-Richards two-state exchange model:^18,27^

1 *R*_2eff_ = *R*_20_ + *R*_ex_(1/*τ*)

in which2
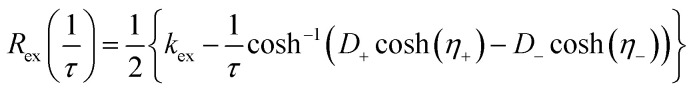
3
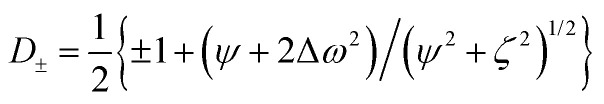
4
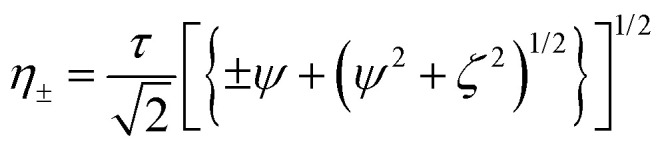
and *ψ* = *k*_ex_^2^ − Δ*ω*^2^, *ζ* = −2Δ*ωk*_ex_(1 − 2*p*_F_), Δ*ω* is the chemical shift difference between the exchanging states, *R*_20_ is the average limiting value of the relaxation rate constant for processes other than chemical exchange, *p*_F_ is the population of the minor (less populated) free (apo) state, which is related to the major, bound state *p*_F_ = 1 – *p*_B_, and *τ* = 1/(2*ν*_cp_) is the spacing between refocusing pulses in the CPMG train. *k*_ex_ = *k*_off_ + *k*_on_[*L*] is the sum of the off- and on-rates, and [*L*] is the concentration of free ligand. *k*_ex_ can be expressed alternatively as: *k*_ex_ = *k*_off_/*p*_F_.

Individual data points were weighted by their estimated uncertainties during the fitting process. The statistical significance of each fit was assessed by also fitting the data to a constant *R*_20_ value (*i.e.* modelling a flat dispersion profile, indicating the absence of exchange), and the *F*-test was used to discriminate between models by rejecting the simpler model at the level *p* < 0.001. Errors in the fitted parameters were estimated from 1000 synthetic data sets created using Monte-Carlo simulations.^39^

### Linear free energy relationships

Linear free energy relationships correlate the change in the free energy of the transition state with those of the free and bound ground states upon a perturbation of the system, which in the present case is introduced as a change in the ligand structure. The response of the free energy of the transition state upon perturbation is expected to be intermediate to those of the ground states, such that it can be expressed as a linear combination of the free-energy changes of the ground states:^21^5
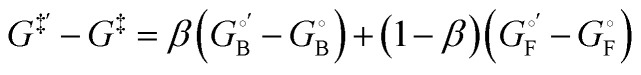
where *G*^‡^, *G*_F_°, and *G*_B_° are the free energies of the transition state, free ground state, and bound ground state, respectively, and *β* (0 ≤ *β* ≤ 1) captures the extent to which the transition state resembles the bound state. Primed and unprimed variables correspond to the perturbed and non-perturbed system, respectively. Using 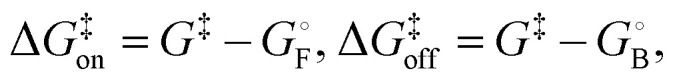 and 
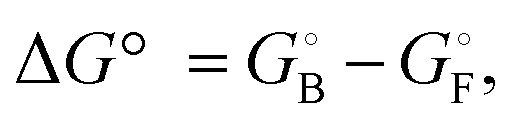
eqn (5) can be rearranged to yield:6
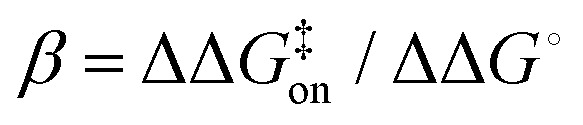
or, alternatively7
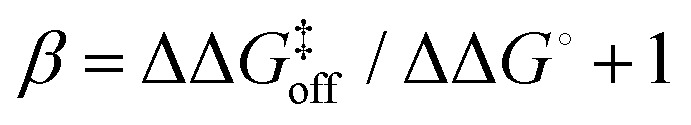
where ΔΔ*G*° = Δ*G*°′ − Δ*G*°, *etc.* More generally, when correlating the effect of several perturbations (*i.e.*, the kinetic and equilibrium binding of several ligands), eqn (6) and (7) can be expressed as8
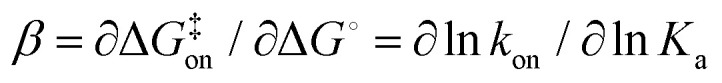
9
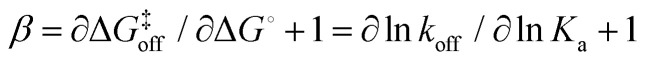
thereby identifying *β* as the slope of the LFER between the free energy barrier of ligand binding and the standard free energy of binding. The second equality in eqn (8) and (9) is valid under the assumption that pre-exponential factors (*e.g.*, the transmission coefficient) of the rate constants are similar for the all perturbed systems. This assumption is expected to hold in the present case given the similarity of the ligands.

## Conflicts of interest

There are no conflicts to declare.

## Supplementary Material

CB-002-D0CB00229A-s001
