# Supplementary material for: Mapping the energy landscape of protein–ligand binding via linear free energy relationships determined by protein NMR relaxation dispersion
Source: RSC Chem Biol. 2020 Dec 23;2(1):259–65. doi: 10.1039/d0cb00229a (PMC8341105; doi:10.1039/d0cb00229a)
Supplement: CB-002-D0CB00229A-s001 [file CB-002-D0CB00229A-s001.pdf]

Electronic Supplementary Material (ESI) for RSC Chemical Biology.
This journal is © The Royal Society of Chemistry 2020

Electronic Supplementary Information

Mapping the Energy Landscape of Protein-Ligand Binding Via Linear Free Energy Relationships Determined by Protein NMR Relaxation Dispersion

Olof Stenström, Carl Diehl, Kristofer Modig, Ulf J. Nilsson, and Mikael Akke*

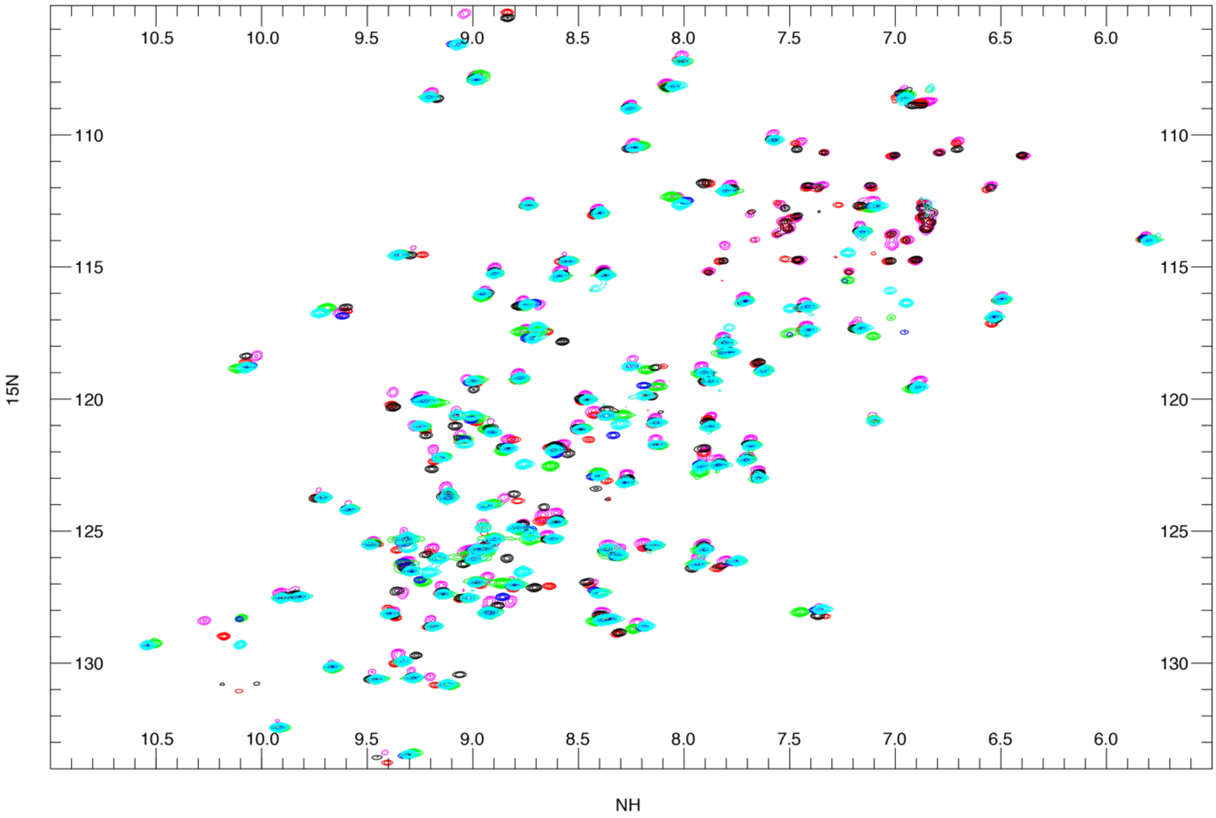

Figure S1. ^{1}H-^{15}N HSQC spectra of apo-galectin-3C and the five different ligand–galectin-3C complexes. Black, apo; Red, L2; Magenta, L3; Blue, oL4; Cyan, mL4; Green, pL4. The spectra were acquired with different spectral widths in the ^{15}N dimension, which leads to slightly different peak positions for peaks that are folded into the spectrum, including side-chain groups of arginine, histidine, and lysine.

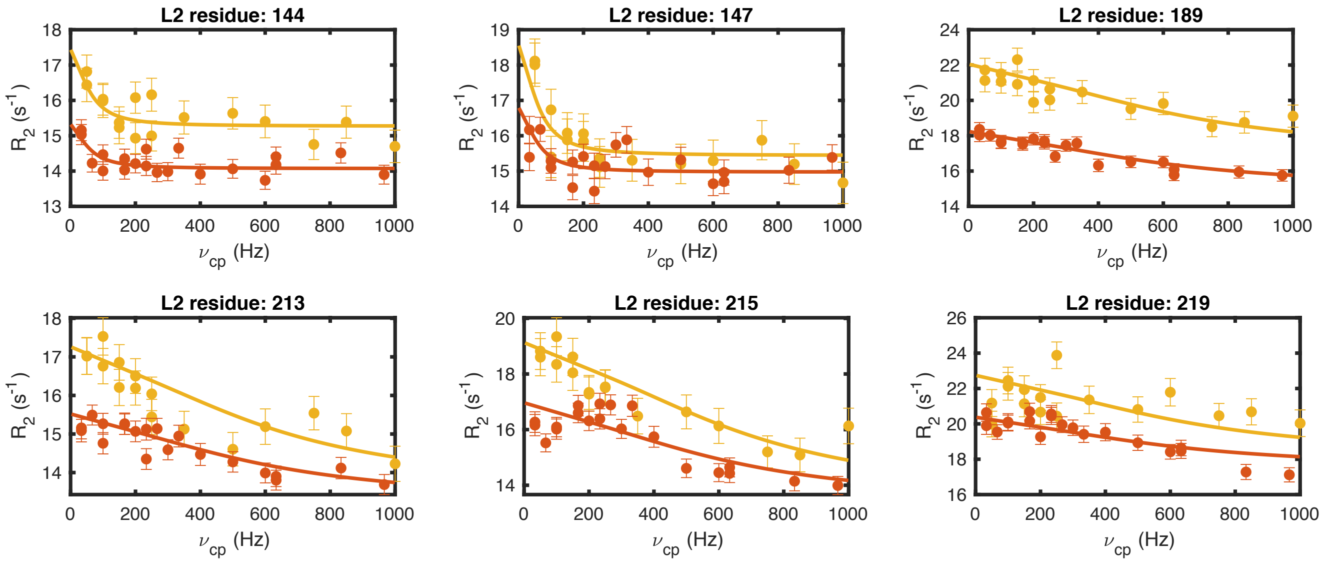

Figure S2. Relaxation dispersion data for all residues showing a significant dispersion in the L2–galectin-3C complex: R144, L147, V189, V213, D215, and L219. Red and orange symbols represent data obtained at static magnetic field strengths of 14.1 T and 18.8 T, respectively.

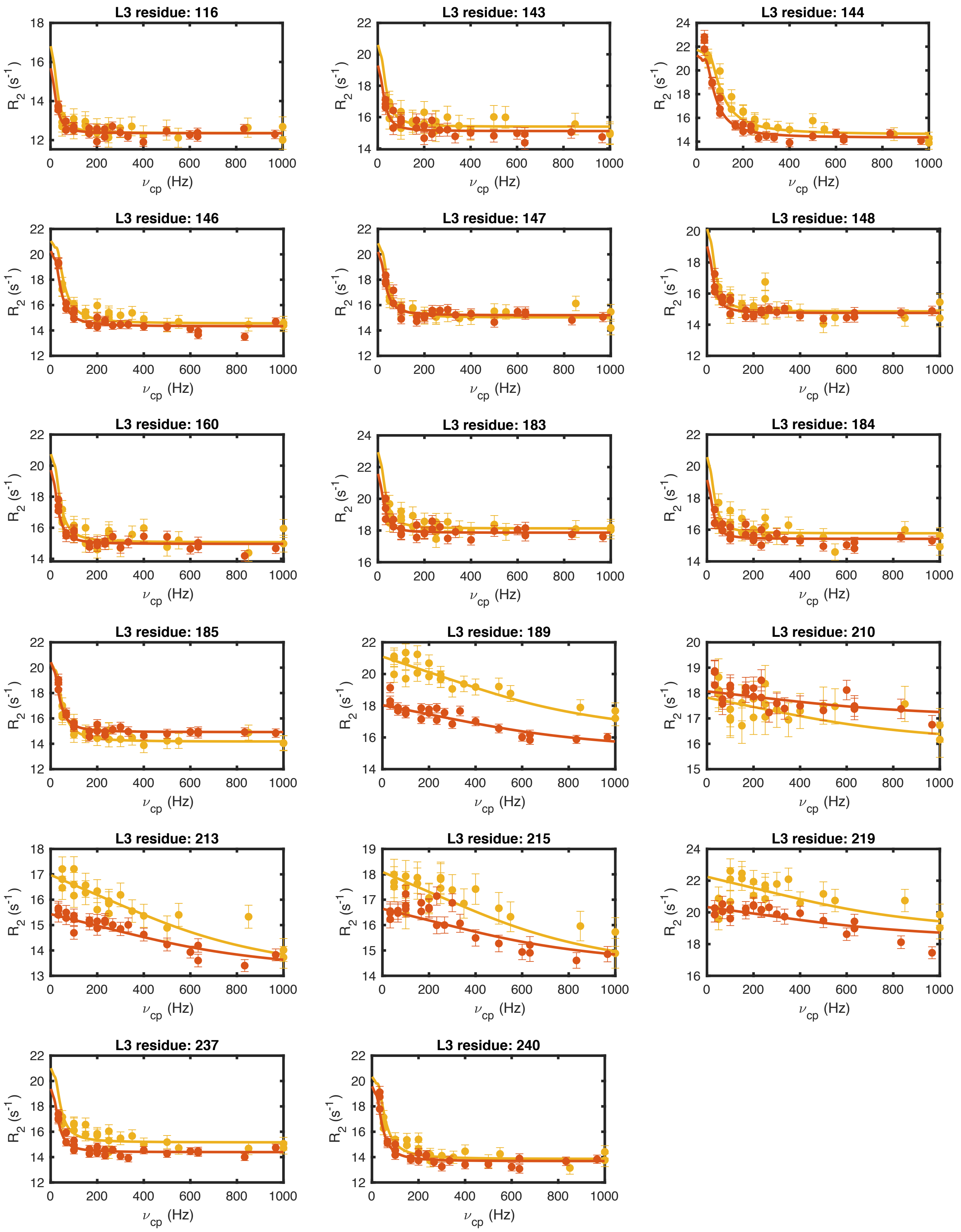

Figure S3. Relaxation dispersion data for all residues showing a significant dispersion in the L3–galectin-3C complex: V116, N143, R144, A146, L147, D148, N160, R183, E184, E185, V189, K210, V213, D215, L219, S237, and I240. Red and orange symbols represent data obtained at static magnetic field strengths of 14.1 T and 18.8 T, respectively.

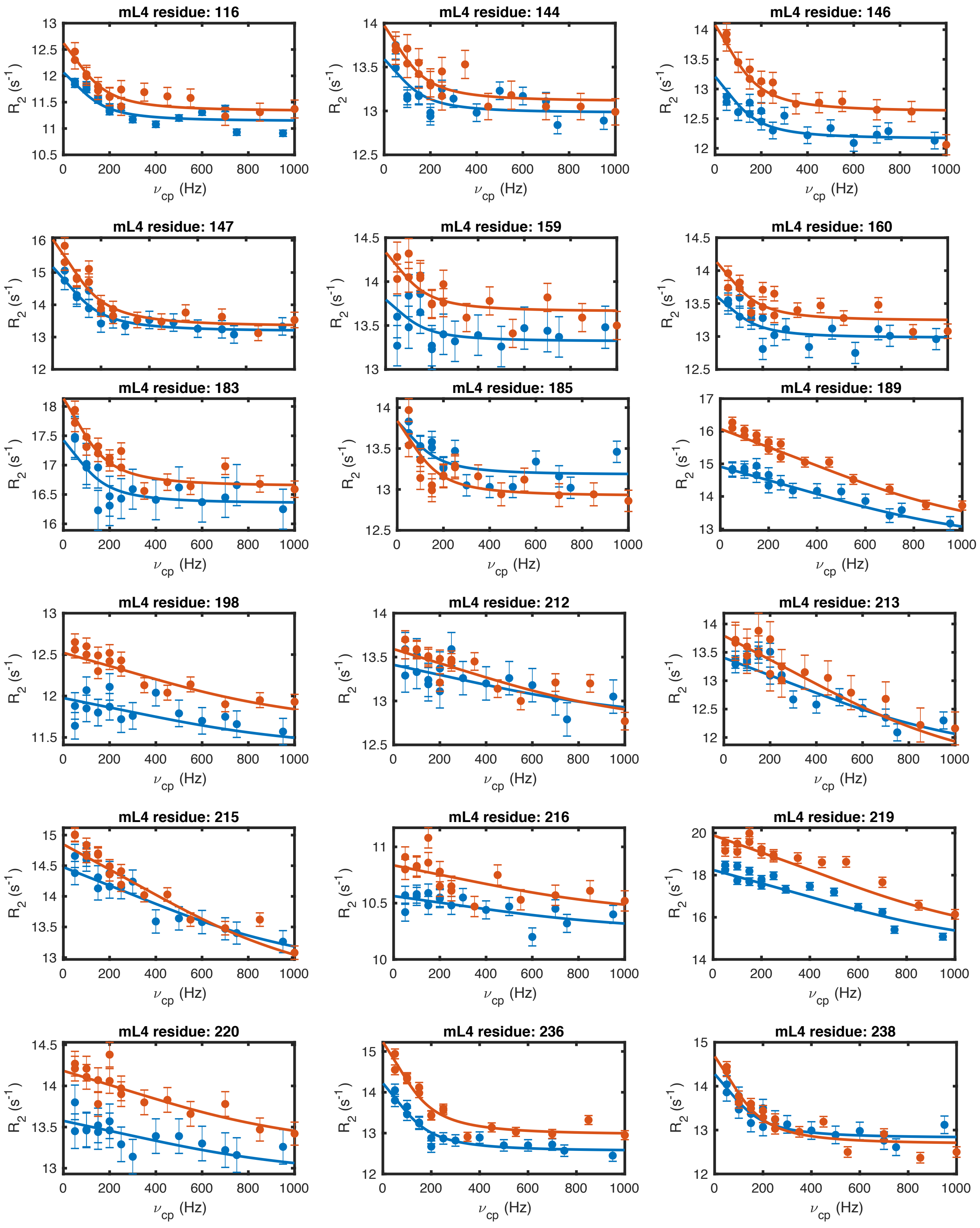

Figure S4 continues on next page.

Figure S3, continued:

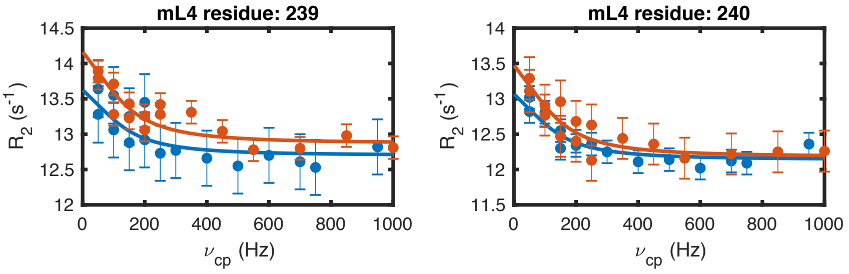

Figure S4. Relaxation dispersion data for all residues showing a significant dispersion in the mL4–galectin-3C complex: V116, L147, V170, T175, K210, A212, V213, D215, L219, Q220, G238, and D239. Blue and red symbols represent data obtained at static magnetic field strengths of 11.7 T and 14.1 T, respectively.

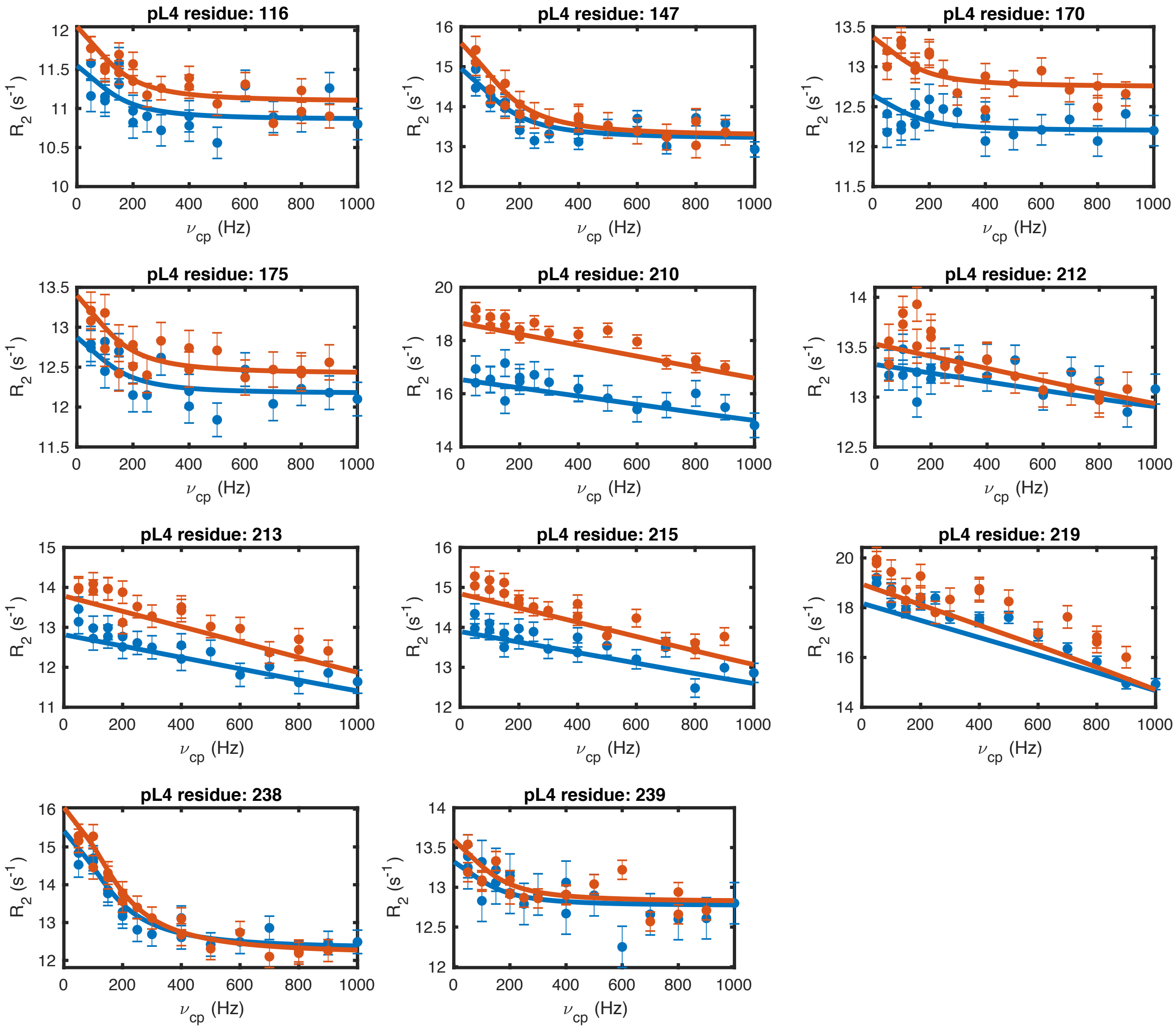

Figure S5. Relaxation dispersion data for all residues showing a significant dispersion in the pL4–galectin-3C complex: V116, R144, A146, L147, F159, N160, R183, E185, V189, F198, A212, V213, D215, A216, L219, Q220, I236, G238, D239, and I240. Blue and red symbols represent data obtained at static magnetic field strengths of 11.7 T and 14.1 T, respectively.

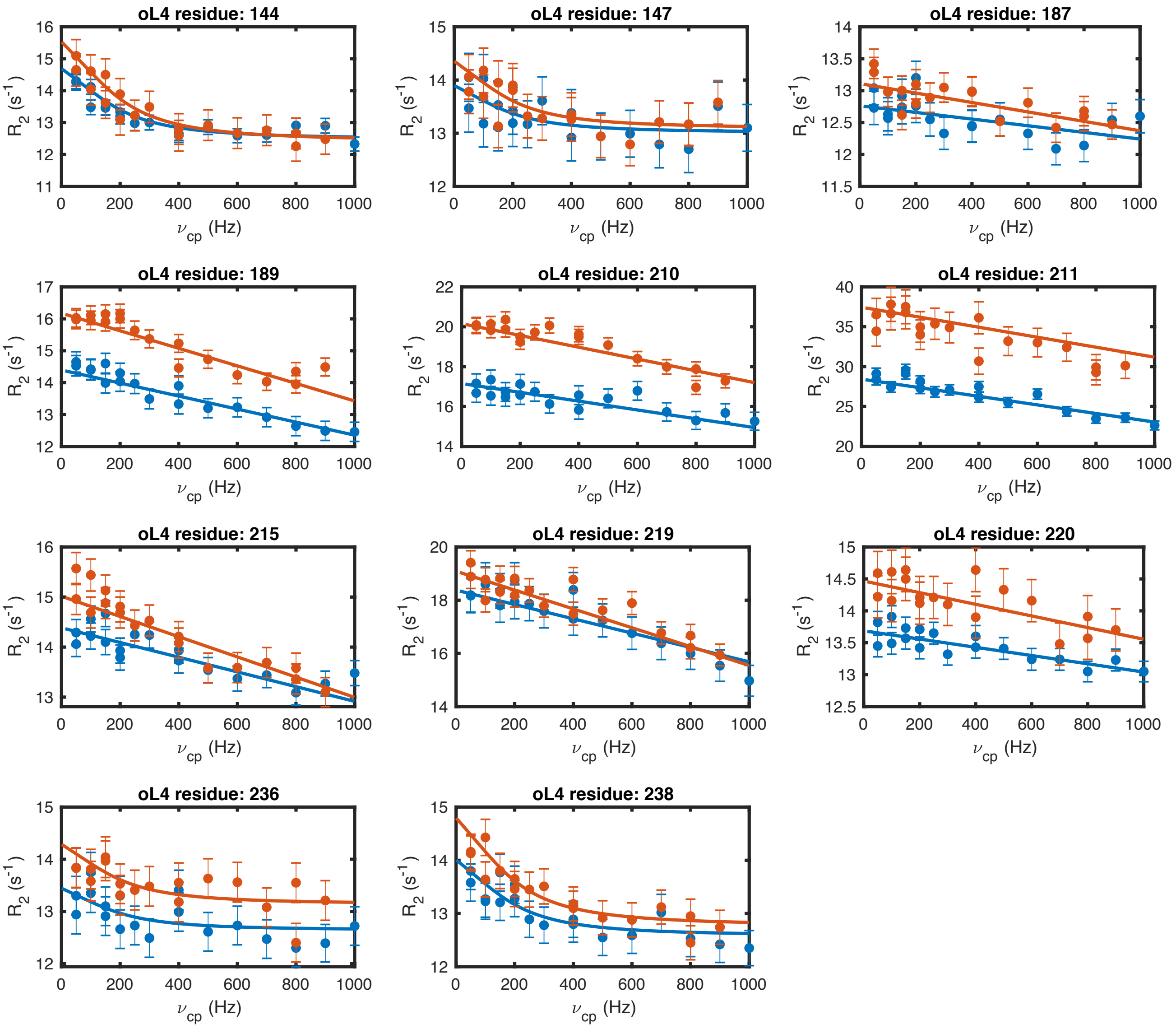

Figure S6. Relaxation dispersion data for all residues showing a significant dispersion in the oL4–galectin-3C complex: R144, L147, Q187, V189, K210, V211, D215, L219, Q220, I236, and G238. Blue and red symbols represent data obtained at static magnetic field strengths of 11.7 T and 14.1 T, respectively.

A

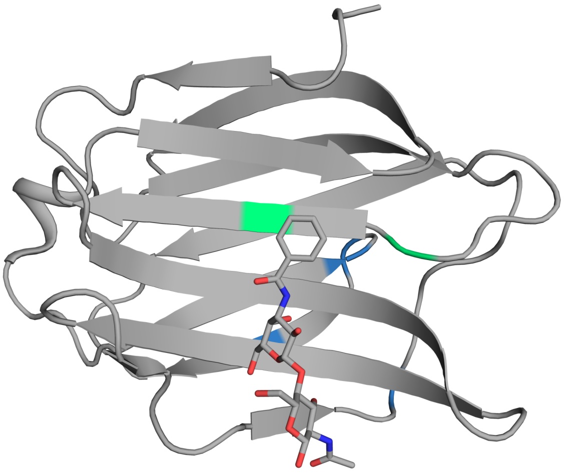

B

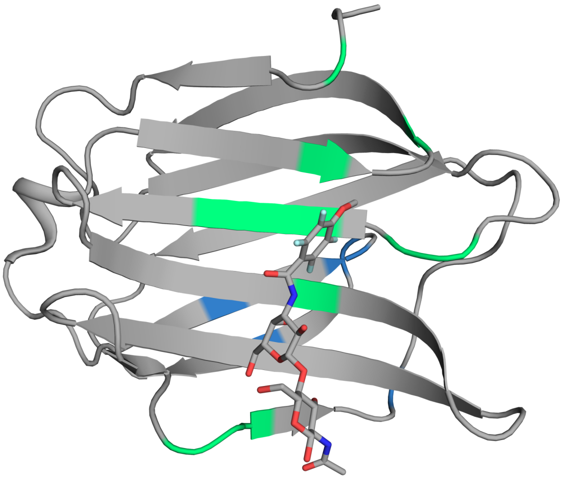

C

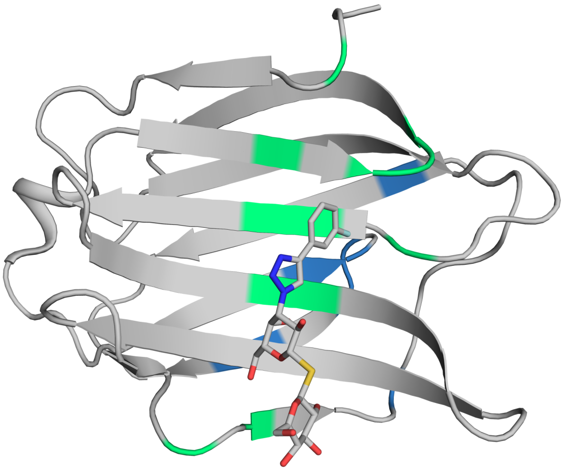

D

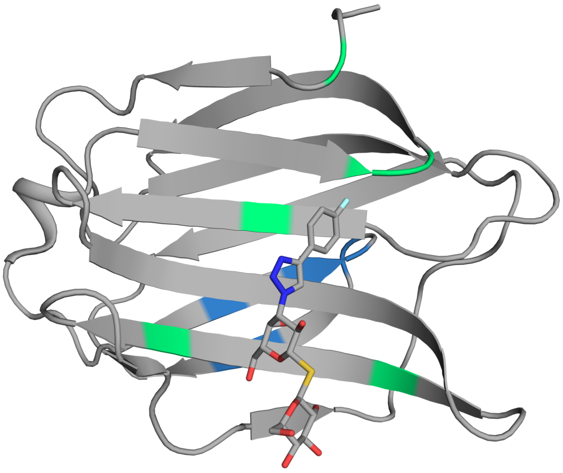

E

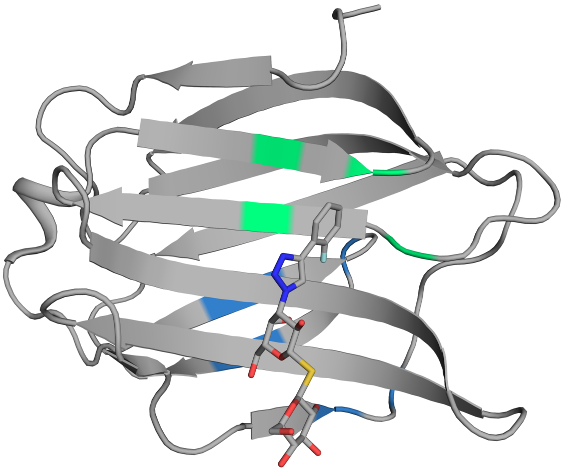

Figure S7. Crystal structures of the five ligand–galectin-3C complexes. The backbone trace is shown in grey ribbon representation with residues exhibiting significant relaxation dispersions highlighted. Green indicates residues reporting on ligand binding kinetics, while blue indicates residues showing fast conformational exchange. Panels A to E (top to bottom): A, L2 (PDB entity 2XG3); B, L3 (1KJR); C, mL4 (6RZG); D, pL4 (6RZH); and E, oL4 (6RZF). The ligands are shown in stick representation with the carbon atoms colored grey, nitrogens blue, oxygen red, sulfur yellow, and fluorine pale blue. The figure was prepared using PyMOL (Schrödinger, LLC).

Table S1. Fitted residues and resulting exchange parameters.

Ligand	Group[a]	Grouped Residues	k_{ex} (s^{-1})	Population[b]
L2	1	R144, L147	385 ± 126	0.93 ± 0.04
	2	V189, V213, D215, L219	5370 ± 850	N.A.
L3	1	V116, N143, R144, A146, L147, D148, N160, R183, E184, E185, S237, I240	123 ± 20	0.94 ± 0.01
	2	V189, K210, V213, D215, L219	4110 ± 760	N.A.
pL4	1	V116, L147, V170, T175, G238, D239	859 ± 115	0.99 ± 0.01
	2	K210, A212, V213, D215, L219, Q220	8386 ± 691	N.A.
mL4	1	V116, R144, A146, L147, F159, N160, R183, E185, I236, G238, D239, I240	808 ± 55	0.98 ± 0.01
	2	V189, F198, A212, V213, D215, A216, L219, Q220	4804 ± 448	N.A.
oL4	1	R144, L147, I236, G238	1210 ± 209	0.97 ± 0.01
	2	Q187, V189, K210, V211, D215, L219, Q220	15800 ± 386	N.A.

[a] Groups of residues fitted jointly to the exchange model.

Group 1: slow exchange corresponding to ligand binding/unbinding.

Group 2: fast conformational exchange independent of ligand binding/unbinding.

[b] N.A., The population cannot be determined reliably under fast exchange conditions.
